# Out of sight, out of mind? How discarded items shape environmental judgments

**DOI:** 10.1186/s41235-026-00743-9

**Published:** 2026-06-28

**Authors:** Emil Skog, John E. Marsh, Patrik Sörqvist

**Affiliations:** 1 Department of Health, Learning and Technology, Luleå University of Technology, Luleå, Sweden; 2 Human Factors Laboratory, School of Psychology and Computer Sciences, University of Central Lancashire, Preston, UK; 3 Faculty of Society and Design, Bond University, Gold Coast, Australia; 4 Department of Building Engineering, Energy Systems and Sustainability Science, University of Gävle, Gävle, Sweden

**Keywords:** Context effect, Contrast, Assimilation, Ecological footprint, Food products

## Abstract

Consumers often inspect products before making purchase decisions. Some items are selected for their shopping baskets, while others are discarded. Can discarded products influence how the selected set is perceived? We examined this question across two experiments. Participants viewed sequences of grocery products, some included in the shopping basket and some discarded, and were asked to provide a summative estimate of the included set’s environmental friendliness after presentation. In Experiment 1, participants had to remember which items to consider for the upcoming judgment, as each item disappeared after presentation. In Experiment 2, each item remained visible after presentation. Discarded green (environmentally friendly) items produced a downshift in perceived environmental friendliness of the included set of items in Experiment 1 (a contrast effect), but an upshift in Experiment 2 (an assimilation effect). Correspondingly, discarded red (environmentally harmful) items produced an upshift in Experiment 1 and a downshift in Experiment 2. These findings show that discarded items can shift judgments of environmental friendliness, but that the direction of this effect depends on whether discarded items are mentally excluded or perceptually integrated with the remaining set.

**Supplementary Information:**

The online version contains supplementary material available at 10.1186/s41235-026-00743-9.

## Introduction

Consumers frequently inspect products before deciding which ones to purchase, carefully selecting some while discarding others. Environmentally conscious shoppers, for instance, may prioritize items labeled as ‘organic’ and ‘low carbon footprint’ while rejecting less sustainable alternatives. When later evaluating the ecological footprint of their purchases, these eco-labels serve as salient reference points (Rondoni & Grasso, 2021; Thøgersen, 2000). However, an overlooked question is whether discarded items also influence judgments of one’s own purchases, even though discarded items are not included in the final purchase. Could products that were considered but ultimately rejected shape perceptions of the selected set? To evaluate this question, the present study focuses on how a discarded eco-labeled item can act as a contextual reference point.

Judgments of environmentally significant information are malleable and prone to biases (Holmgren et al., 2018; Kim & Schuldt, 2018; Luo & Zhao, 2021; Sörqvist et al., 2020; Tanner & Jungbluth, 2003; Zhao & Luo, 2021). Context can exert a strong influence on perception and judgment (Bless & Schwarz, 2010; Evangelidis et al., 2024), and environmentally significant information can be perceived differently depending on anchors and related reference points (Joireman et al., 2010; Skog & Sörqvist, 2025; Skog et al., 2025). A discarded eco-labeled item may therefore alter perception of the remaining set of included items, if it can act as a contextual reference point. While the present study investigates a potential shift in perception, this may have implications for consumers’ environmentally responsible behavior, as behavior often depends on how products are seen and evaluated. Related behavioral effects are moral licensing (e.g., Mazar & Zhong, 2010) and rebound effects (e.g., Seebauer, 2018), where perception of one’s own environmental responsibility can influence future choices. For example, a built-up sense of high environmental responsibility can construct a moral license to act unsustainably, and vice versa. One’s sense of environmental responsibility may be influenced by choices to include or discard eco-labeled items in purchases.

Context can have different effects, which in part depends on how information is integrated (Tanner, 2008; Wedell et al., 1987). One type of context effect in human judgment is assimilation (Brown et al., 1992; Martin et al., 1990; Meyers-Levy & Sternthal, 1993; Poulton, 1979; Schwarz & Bless, 1992)—the tendency for judgment of attribute values (e.g., judgment of carbon footprints; Skog et al., 2026a) to be drawn toward the attribute values of other, related but non-target stimuli. For example, target faces are judged as less attractive when surrounded by less attractive faces and more attractive when surrounded by more attractive faces (Wedell et al., 1987), even though the surrounding faces are not to-be-estimated stimuli. Similarly, when products are evaluated side by side, judgment of a specific product’s environmental friendliness tends to be assimilated with the concurrent products’ environmental friendliness (Tanner, 2008). A product may appear more environmentally friendly in the presence of other, highly environmentally friendly products, and less environmentally friendly in the presence of other, environmentally harmful products.

Context can also produce a contrast effect—the opposite of assimilation. For example, a specific face in a set of sequentially presented faces can appear more attractive if the rest of the set predominantly comprises unattractive faces (Wedell et al., 1987). Thus, the perceived attractiveness of the face is pushed away from the attribute values of the contextual faces. Similarly, when products are judged separately on their environmental friendliness, judgment of a specific product’s environmental friendliness tends to be pushed away from the environmental friendliness of other products (Tanner, 2008).

Whether assimilation or contrast occurs therefore seems to depend on the characteristics of the context (Barker & Imhoff, 2021; McKenna, 1984) and presentation format (Tanner, 2008; Wedell et al., 1987), with simultaneous presentation tending to produce assimilation and sequential presentation tending to produce contrast. A theoretical account of assimilation and contrast effects is offered by the inclusion/exclusion model (Bless & Schwarz, 2010; Schwarz & Bless, 1992). According to this model, if a target stimulus and its context are mentally grouped together, assimilation effects occur, leading to judgments that are drawn toward the contextual reference. For example, if an environmentally harmful (red labeled) product is perceived as part of the same shopping basket as eco-friendly items, an assimilation effect should occur. Perceived commonality between target and context items can strengthen assimilation effects in carbon footprint judgments (Skog et al., 2026a). However, if an environmentally harmful product is mentally excluded from the representation of the target, a contrast effect should emerge. If an environmentally harmful product is excluded from the shopping basket, the remaining content of the basket may instead appear more eco-friendly, as the environmentally harmful product provides contrast. When this happens, discarded items serve as reference points that shift judgments in the opposite direction. Discarding an item triggers a subtraction process, whereby the valence of the discarded item is mentally removed from the judgment of the remaining items. A similar process has been observed in social evaluations: for example, if people learn that a respected politician has left a political party, their perception of the party’s respectability diminishes (Schwarz & Bless, 1992).

In the present paper, we take the novel step to explore how exclusion of shopping products with high or low environmental valence produces contrast or assimilation effects in judgment of the set of included items, and how this may depend on presentation format (sequential versus simultaneous presentation, in Experiments 1 and 2, respectively). If consumers view a sequence of products and select some for inclusion in their basket and discard others, the mental exclusion of the discarded products might be particularly strong if the discarded products are out of sight. With sequential presentation of included and discarded items (i.e., a presentation format in which each item disappears after presentation), exclusion should then produce contrast to judgments of the environmental friendliness of the remaining, included set. This was the main hypothesis of Experiment 1. As the sequential presentation of the shopping basket was hypothesized to influence the contrast effect, we introduce Experiment 1 with a rationale for testing sequence effects (e.g., where in the sequence an eco-labeled item would appear, and how long the sequence was). But before turning to Experiment 1, we report an exploratory pilot experiment.

## Pilot experiment

We began the empirical work by conducting an exploratory pilot experiment. The pilot experiment is reported in full in the Supplementary Materials, with method, results and data visualization. The aim of the pilot experiment was to develop and test the general experimental procedure, in preparation for Experiment 1, and to obtain an initial measure of a contrast effect which could be used to inform a power analysis for Experiment 1. Data from the pilot experiment are publicly available online at the Open Science Framework (OSF; 10.17605/OSF.IO/XH5EA). The results of the pilot experiment provided a preliminary indication of a contrast effect, in which shopping baskets (i.e., stimulus sequences) were judged as more environmentally friendly when a red item was discarded during sequence presentation, compared to when a green item was discarded. A targeted analysis on this possible contrast effect estimated an effect size of *d*_z_ = .296, classified as small-medium. This result was used to inform the power analysis of Experiment 1.

## Experiment 1

Discarding an item may alter the perceived environmental friendliness of the contents of a basket, even though a discarded item does not belong within the basket’s content. If a ‘high carbon footprint’ label is *included* in the basket, we expect a less favorable environmental evaluation. But centrally, based on the inclusion/exclusion model of assimilation and contrast (Bless & Schwarz, 2010), we predict a reversed effect when such an item is *excluded*: Discarding a high carbon footprint product should make the remaining selection appear more environmentally friendly, whereas discarding an eco-friendly item should have the opposite effect, making the contents of the basket seem less sustainable. Here, discarded products might act as a standard for comparisons when the remaining contents of the basket is judged. The primary aim of Experiment 1 was to find this hypothesized contrast effect from discarded items, using a sample size informed by the pilot experiment, with a new design that was developed based on the pilot experiment.

Experiment 1 also investigated sequence effects as the presentation format of the shopping basket might influence the measured effects. We tested the effects of ‘sequence type’, where ‘primacy’ and ‘recency’ sequences placed the eco-labeled item first or last in the stimulus sequence, respectively. In the present study, we manipulated the position of the eco-labeled item to explore whether this influences the hypothesized contrast effect. Experiment 1 imposed a memory demand on participants, as items in the unfolding sequence were presented one at a time. If some items are recalled better than others, this may influence the judgment formation process and the contrast effect. We have previously demonstrated that environmental judgments are biased by a recency effect (Sörqvist et al., 2024) that appears based on the ability to recall the items (Sörqvist et al., 2025). One other possibility is a scale-adjustment or criterion-setting account, whereby an early eco-labeled item establishes a reference point against which the rest of the basket is evaluated (Eiser, 1990; Frederick & Mochon, 2012; Wedell, 1996). In this view, the first eco-labeled item may anchor the perceived environmental standard for the sequence, with this criterion persisting across subsequent items. This predicts that early eco-labeled items may exert a greater influence than late items in the sequence. Such an account provides a potential mechanism for primacy effects that is distinct from memory accessibility accounts typically used to explain recency effects.

In a further development of our investigation of sequence effects, Experiment 1 also used sequences of both five items (as in the pilot) and ten items. We speculated that, when sequences extend in length, the final item exerts a greater influence on judgment, likely because a longer sequence facilitates the formation of a more stable mental representation (Cowan et al., 1993). If contrast effects emerge due to a comparison between the final item and the rest of the sequence, then a longer sequence might accentuate the contrast effect by making deviations from the rest of the sequence more salient. People may also rely more on an eco-labeled final item (which may be more accessible in memory; Sörqvist et al., 2025) when lists are longer and thus more difficult to accurately recall. Given the possible effects of list length, Experiment 1 introduced sequence length as a manipulation to examine whether a longer sequence would modulate the strength of the contrast effect.

Experiment 1 was structured as a 2 (Color: green and red) × 2 (Sequence type: primacy and recency) × 2 (Cue: include and discard) × 2 (Sequence length: five items and ten items) within-participants factorial design. This design allowed a test of the contrast effect (which was our primary hypothesis), while also comparing recency vs. primacy effects and different sequence lengths.

## Method

### Participants

The sample size was determined through an a priori power analysis using G*Power (Faul et al., 2007), based on the effect size of *d*_z_ = .296 obtained from the pilot experiment. Although the overall design included multiple within-participants factors, our primary hypothesis concerned a specific pairwise contrast between discard conditions (discarding a red versus a green eco-labeled item). Power estimation was therefore based on paired-samples effect sizes (Cohen’s *d*_z_) corresponding to these planned contrasts, rather than on omnibus interaction effects. Omnibus ANOVAs were used to characterize the broader pattern of sequence effects but were not the basis for sample size determination. Based on this estimate, a sample size of *N* = 92 was required to achieve 80% power (1–*β* = .80) to detect the hypothesized contrast effect with discarded items. To meet this requirement, we recruited 92 participants via Prolific (44 female, 48 male, Mean age = 42.9 years, *SD* = 14.5), ensuring that none had participated in the pilot. The ethical screening and procedure adhered to the Declaration of Helsinki and the guidelines by the Swedish Research Ethics Authority guidelines (Dnr 2024–05795-01). Participants were compensated at a rate of £9 per hour, with the experiment lasting approximately 31 min.

### Materials

The experiment was programmed in PsychoPy (version 2024.1.0, Peirce et al., 2019) and deployed online using Pavlovia. Participants accessed the experiment via a Prolific URL and were required to use a desktop computer.

Stimuli consisted of 81 images of grocery store products (e.g., packages of rice, meat, and fruit). Each image was displayed above a text label describing the item (e.g., ‘1 bunch of bananas’). The items were used to construct four sequence types: green eco-labeled sequences (where a green-labeled item appeared in the first or last position) and red eco-labeled sequences (where a red-labeled item appeared in the first or last position). At presentation, the products were presented randomly (without any product appearing more than once within the same sequence). Thus, the same product could (by chance) be combined with a green label in one trial and a red label in another trial. Each sequence contained five or ten items, and item selection was randomized on each trial to prevent systematic pairing biases between specific products and eco-labels.

### Design and procedure

The study was advertised on Prolific as an investigation of ‘Environmental impact and grocery shopping’. Before starting the experiment, participants read a participant information sheet, provided informed consent, and received detailed task instructions. They were informed that they would view sequences of grocery store products, with some items labeled green (eco-friendly) or red (high carbon footprint). Participants were instructed that each product would be followed by a cue indicating whether it should be included or discarded from their shopping basket. They were told only to consider the included items in their basket when making environmental impact judgments. Participants were explicitly instructed to evaluate each shopping basket independently, without considering previous trials’ baskets.

**Fig. 1:**
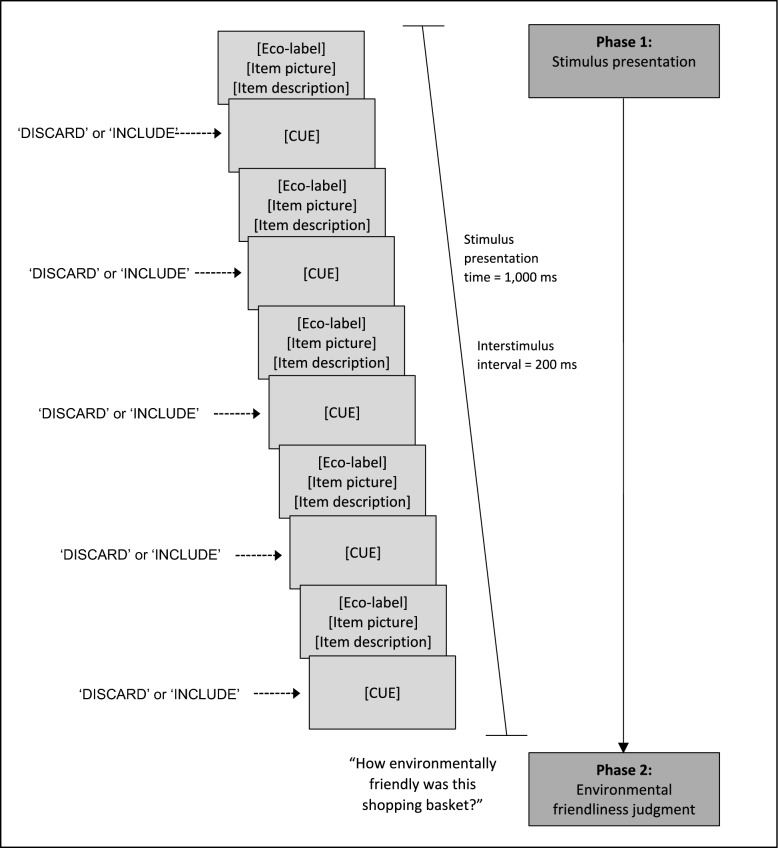
Trial structure in Experiment 1: Each item was followed by a cue (either ‘DISCARD’ or ‘INCLUDE’) that told the participant whether to include or discard the item from their to-be-rated shopping basket. Half of the trials in Experiment 1 had sequences with 5 items, as in the figure. The other half had 10 items, with identical item-wise structure

The trial structure was inspired in part by the item-wise directed forgetting literature (Basden, 2010; MacLeod, 1999). Each trial began when participants pressed the spacebar button. Thereafter, a sequence of five or ten items was presented one at a time in the center of the screen. See Fig. 1 for an illustration of the trial structure. Each item was displayed for 1000 ms, followed by a 200 ms blank interval. A cue ‘INCLUDE’ or ‘DISCARD’ then appeared for 1000 ms, followed by another 200 ms blank interval before the next item. The total stimulus sequence duration was 12 s for sequences with five items and 24 s for sequences with ten items. Participants were not informed in advance about the sequence length for each trial. After viewing the stimulus sequence, participants rated the environmental friendliness of their shopping basket using a 9-point scale (1 = not environmentally friendly and 9 = very environmentally friendly), which ended the trial.

We used a fully within-participants design, where all participants completed trials in all 16 conditions (2 × 2 × 2 × 2). Experiment 1 comprised a factorial design with four factors: color (green and red), sequence type (primacy and recency), cue (include and discard), and sequence length (five items or ten items). Exactly one item in each sequence had an eco-label (Color: green or red). Sequence type determined whether the eco-labeled item would appear first (primacy) or last (recency) in the sequence. Cue determined whether all items would be included (include) or whether all non-labeled items would be included and the singular eco-labeled item would be discarded (discard). Sequence length determined whether the sequence would contain five (five items) or ten items (ten items). Thus, this rich factorial design constituted 16 conditions, and each condition was repeated four times, totaling 64 trials. The dependent variable was the mean ratings of the four trials per condition. The presentation order of the conditions was randomized, while also ensuring that all conditions were completed before any condition was repeated. Before the main experiment, participants completed three practice trials to familiarize themselves with the task. Responses from these trials were not analyzed. Upon completing the experiment, participants were shown a debrief explaining the study’s objectives.

### Availability of data and transparency

The study’s design, measures, sample size, analyses, exclusion criteria, and results are fully detailed in this report. Statistical analyses were conducted using Jamovi (version 2.3.28.0) and JASP (version 0.18.3). In addition to frequentist analyses, Bayesian analyses were conducted to quantify evidence for effects relative to the null hypothesis. Bayes factors are reported as BF10 values derived from JASP’s analysis of effects framework using default priors. In some cases, Bayes factors reached extremely large values, reported as BF10 = ∞ due to numerical overflow in JASP rather than literal infinite evidence. Bayesian results are interpreted alongside frequentist statistics to aid inference rather than as a replacement for them. This study was not preregistered, but all data and materials (Skog, Marsh, & Sörqvist, 2026b) are available on the Open Science Framework (OSF; 10.17605/OSF.IO/XH5EA).

## Results and discussion

We first report planned comparisons directly testing our primary hypothesis concerning a contrast effect from discarded eco-labeled items. We then report an omnibus repeated measures ANOVA to characterize how sequence effects such as recency influenced environmental judgments.

### Primary hypothesis testing: The contrast effect

**Fig. 2:**
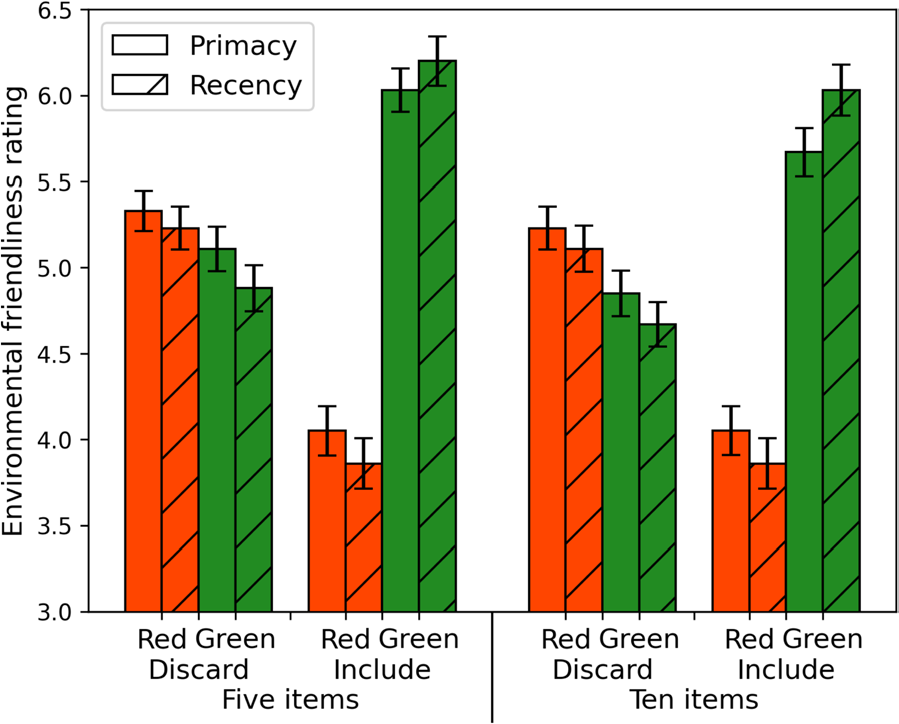
Results of Experiment 1: Data are split across the four experimental factors. Primacy and recency refer to where in the stimulus sequence the eco-labeled item (red/green) and its cue (discard/include) was placed. Error bars represent the standard errors of the means

Our first analysis directly tested for the hypothesized contrast effect with discarded eco-labeled items. In support of the hypothesis, contrast effects occurred for discarded items, in both primacy and recency sequences (Fig. 2). Discarding a red item led to higher environmental friendliness ratings of the remaining basket than discarding a green item in both primacy, *t*(91) = 3.17, *p* = .002, *d*_z_ = .331, Mean difference = 0.302 (95% CI [0.113–0.491]), BF_10_ = 11.942, and recency sequences, *t*(91) = 2.91, *p* = .005, *d*_z_ = .304, Mean difference = 0.394 (95% CI [0.125–0.663]), BF_10_ = 5.943. Bayes factors strongly and moderately favored the hypothesis over the null hypothesis, respectively. This suggests that the basket was compared to the discarded eco-labeled item, which acted as a standard for comparisons. The discarded item provided subtractive contrast which repelled judgments of the basket away from the environmental valence of the discarded item. We found that this effect was the same regardless of sequence length (see Fig. 2), and thus, the above *t*-tests tested the average of the two sequence length conditions (five and ten items).

### Sequence effects: evidence from omnibus analysis of variance

This analysis, of secondary interest, focuses on sequence effects. We explored the effects and interactions that emerged from our rich factorial design with a repeated measures ANOVA with factors: 2 (Color: green and red) × 2 (Sequence type: primacy and recency) × 2 (Cue: include and discard) × 2 (Sequence length: five items and ten items). We first consider some interactions of higher interest, and then, for completeness, report other interactions and main effects. An outcome of particular interest was the significant three-way interaction between color × cue × sequence type, *F*(1, 91) = 11.22, *p* = .001, *η*^2^_*p*_ = .110, BF_10_ = 89.771. The Bayes factor very strongly favored the hypothesis over the null hypothesis. This complex interaction, which we interpret together with the data shown in Fig. 2, reveals a couple of key findings. First, a recency effect was found for included items, where environmental friendliness ratings were more influenced by the valence of the eco-labeled item (green = high in eco-friendliness and red = low in eco-friendliness) if this item appeared last in the sequence than if it appeared first in the sequence. This is likely because the recent item is more temporally salient than the first item (Aldrovandi et al., 2015; Sörqvist et al., 2025). Second, the recency effect was not found for discarded items (Fig. 2). Instead, discarded items produced a contrast effect (previously demonstrated with *t*-tests), which was not larger with recency than primacy sequences. The three-way interaction between color × cue × sequence type is consistent with the possibility that contrast effects and recency effects rely on partially distinct mechanisms, as suggested by the fact that the recency effect was found with included but not discarded items (Fig. 2).

This significant three-way interaction between color × cue × sequence type also qualifies the boundaries of two lower two-way interactions that pertain to the recency effect: color × sequence type, *F*(1, 91) = 8.22, *p* = .005, *η*^2^_*p*_ = .083, BF_10_ = 32.303, and cue × sequence type, *F*(1, 91) = 8.23, *p* = .005, *η*^2^_*p*_ = .083, BF_10_ = 34.842. Bayes factors very strongly favored the hypothesis over the null hypothesis. These lower interactions are best understood in light of the interaction between all three factors (color × cue × sequence type), as the recency effect was active with included items more so than with discarded items, and the direction of the recency effect naturally depends on whether the eco-labeled item was red or green (Fig. 2). We note that sequence length (five vs. ten items) did not modulate this effect further, as indicated by a non-significant four-way interaction, *F*(1, 91) = 0.114, *p* = .737, *η*^2^_*p*_ = .001, BF_10_ = .002. The Bayes factor extremely strongly favored the null hypothesis over the hypothesis. The data look similar on the left- and right-hand sides of Fig. 2, indicating that sequence length did not significantly influence the contrast effect or the recency effect.

For completeness, we also examined additional interactions that emerged from the ANOVA. A significant interaction between color × cue, *F*(1, 91) = 108.58, *p* < .001, *η*^2^_*p*_ = .544, BF_10_ = *∞* (reflecting numerical overflow in JASP rather than a literal infinite Bayes factor), confirmed that the effects of red and green eco-labels depended on whether these were included or discarded. Specifically, when an item was included, green and red labels increased and decreased perceived environmental friendliness, respectively. However, when an item was discarded, this effect was even reversed, as indicated by the contrast effect.

A second interaction which we discuss for completeness is color × sequence length, *F*(1, 91) = 9.17, *p* = .003, *η*^2^_*p*_ = .092, BF_10_ = 4.992. The Bayes factor moderately favored the hypothesis over the null hypothesis. Increasing the sequence length from five to ten items resulted in a greater reduction in environmental friendliness ratings when a green item was present (a decrease of 0.25 units on the 9-point response scale) than when a red item was present (a decrease of only 0.05 units). This pattern suggests that participants attributed more weight to environmentally harmful items than to environmentally friendly ones, in line with the negativity bias—where negative stimuli tend to be more salient and resistant to dilution in larger sets (Rozin & Royzman, 2001). Future studies could further explore whether larger sequence sizes attenuate the influence of eco-friendly labels while leaving the impact of harmful labels unchanged. Together, these findings suggest that eco-label color, cues, and sequence position interact in complex ways to shape environmental judgments. While estimates of included items appear to be driven by recency-based judgmental salience, contrast effects emerged with discarded items. These may operate through partially distinct cognitive mechanisms as contrast is stable across sequence positions.

Finally, we report main effects in the ANOVA, for completeness. We note that no ANOVA main effects were critical for our research aims or hypothesis testing. The results (Fig. 2) revealed significant main effects of color, *F*(1, 91) = 99.44, *p* < .001, *η*^2^_*p*_ = .522, BF_10_ = *∞* (reflecting numerical overflow in JASP rather than a literal infinite Bayes factor), and sequence length, *F*(1, 91) = 11.52, *p* = .001, *η*^2^_*p*_ = .112, BF_10_ = 19.613. Participants considered eco-labels when making their judgments, and they rated longer sequences as slightly less environmentally friendly (mean rating of five items: 5.09; mean rating of ten items: 4.94). Sequence type (primacy vs recency) did not produce a significant main effect, *F*(1, 91) = 2.84, *p* = .095, *η*^2^_*p*_ = .030, BF_10_ = 10.385, although the Bayes factor favored the hypothesis over the null hypothesis. This discrepancy in statistical outcomes between frequentist and Bayesian approaches warranted a paired-samples *t*-test, to evaluate whether primacy and recency differed. The *t*-test suffers less complexity than the four-factor ANOVA, as the Bayesian approach with multiple repeated measures factors can be sensitive (van den Bergh et al., 2023). The test found no difference between the average of all primacy and all recency conditions, *t*(91) = 1.69, *p* = .095, with a BF_10_ = 0.448, which does not favor the hypothesis over the null hypothesis. Cue (include and discard) also produced no significant main effect, *F*(1, 91) = 2.71, *p* = .103, *η*^2^_*p*_ = .029. But in the Bayesian analysis, cue produced a surprising main effect of BF_10_ = ∞ (reflecting numerical overflow in JASP rather than a literal infinite Bayes factor), which is difficult to reconcile with the non-significant frequentist test. However, it is likely that the Bayesian analysis of the effect of cue was misguided by a violation of the assumption of normality (van den Bergh et al., 2023), Shapiro–Wilk’s *W* = .935, *p* < .001. A Wilcoxon-rank test found no difference between the average of all include-cue and all discard cue conditions, *W*(91) = 1709, *p* = .173, suggesting that there was no basic difference between include and discard cue conditions.

The omnibus ANOVA analyzed a complex design with four factors. Both ANOVA versions (frequentist and Bayesian) suffer a limitation where the complexity of the repeated measures design introduces noise, making results less robust. We therefore urge a cautious interpretation of these results, especially where violations of the assumption of normality are reported. In contrast, the primary hypothesis was evaluated with a simple *t*-test which did not suffer a violation of the assumption of normality, demonstrating a novel contrast effect with discarded items.

Although participants were instructed to base their judgments only on included items, it is possible that some participants partially incorporated discarded items into their evaluations. Rather than reflecting simple misunderstanding, such effects may indicate that discarded items functioned as contextual reference points that were difficult to ignore, even when explicitly task-irrelevant. The systematic differences between include and discard conditions nevertheless suggest that participants were sensitive to the instruction, even if discarded items continued to exert an unintended influence on judgment.

## Experiment 2

With sequential presentation, Experiment 1 found that discarded items produced contrast effects in judgment of the remaining set. The mental exclusion of discarded products may produce contrast when they are out of sight. Experiment 2 tested whether discarded items would continue to produce contrast when they remained visible at the time of judgment.

Previous research suggests that presentation format modulates contrast and assimilation effects, with sequential presentation tending to produce contrast and simultaneous presentation tending to produce assimilation effects (Tanner, 2008; Wedell et al., 1987). Thus, Experiment 2 aimed to test whether discarded items produce contrast even when they remain visible at the time of judgment. One possibility is that participants adjust their responses following the discard cue, which could still produce contrast. Another possibility is that discarded items produce an assimilation effect under this presentation format, such that the environmental valence of the discarded item pulls judgments of the remaining set in the same direction, even though the included items are identical.

Experiment 2 was structured as a 2 (Color: green and red) × 2 (Sequence type: primacy and recency) × 2 (Cue: include and discard) within-participants factorial design. This design allowed a test of the contrast effect, while also comparing sequence type effects. Given that simultaneous presentation does not impose a memory demand, we did not expect a memory-based recency effect in Experiment 2. However, we retained this factor to match the design of Experiment 1 and to investigate the scale-adjustment hypothesis that an eco-labeled item presented early may act as an anchor for subsequent judgments (Eiser, 1990; Frederick & Mochon, 2012; Wedell, 1996).

Before turning to Experiment 2, it is important to clarify that several methodological features differed between Experiments 1 and 2. Most notably, Experiment 1 used sequential presentation with disappearing items, whereas in Experiment 2 items remained visible at the time of judgment. In addition, the experiments differed in list length, the timing and placement of the include/discard cues, the visual display of the basket, and the presence of a baseline condition. As a result, any differences in observed effects between experiments cannot be attributed uniquely to presentation format. Instead, Experiment 2 should be interpreted as a conceptual extension testing whether the pattern observed in Experiment 1 generalizes under conditions that may promote integration of all items into a unified representation.

## Method

### Participants

We made a new estimate of sample size based on the contrast effect for primacy items in Experiment 1, where discarding a red item led to higher ratings of environmental friendliness for the remaining basket than discarding a green item, with a Cohen’s *d*_z_ = .331. Because our primary hypothesis concerned this specific, pairwise contrast between discard conditions, power estimation was based on paired-samples effect sizes (*d*_z_) corresponding to this planned comparison rather than on omnibus interaction effects. An a priori estimate of sample size with G*Power found that a sample size of *N* = 74 was required to achieve 80% power (1–*β* = .80). We recruited 74 participants via Prolific (38 female, 36 male, Mean age = 45.2 years, *SD* = 13), applying the same procedures for recruitment and ethics as before. Participants were compensated at a rate of £9 per hour for approximately 14 min of total study time.

### Materials, design and procedure

The materials were the same as those of Experiment 1. Each sequence contained seven unique grocery items, randomly selected on each trial. In conditions where one of these items had a green or red eco-label, the pairing of item and eco-label was also random.

**Fig. 3:**
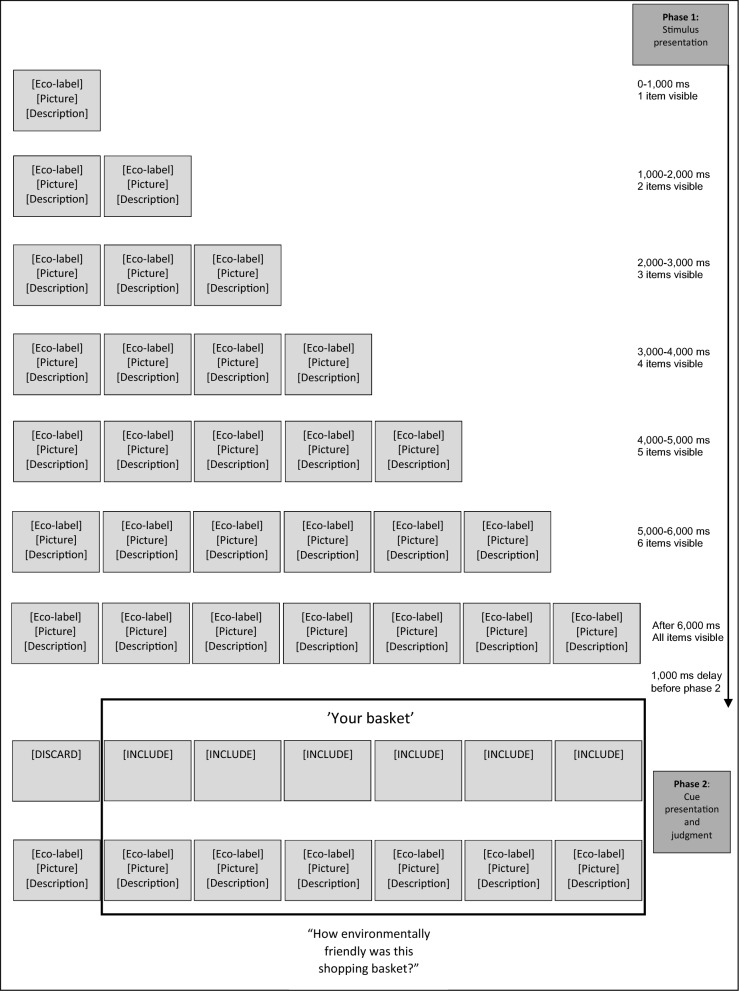
Trial structure in Experiment 2: Items appeared left-to-right, with one new item appearing each second until seven items were shown. When all items had been shown, ‘INCLUDE’ or ‘DISCARD’ cues were presented together with a visual representation of the ‘shopping basket’, capturing the included items within a black rectangle

The general design and procedure were like Experiment 1, where participants received similar instructions on grocery product sequences and eco-labels and performed a similar rating task of judging the environmental friendliness of items included (and only those included) in the shopping basket. But in Experiment 2, the stimulus sequences were altered such that the items remained visible during the stimulus presentation sequence, and until participants gave a rating response. See Fig. 3 for an illustration of the trial structure. One item appeared at a time with one second intervals between them. The first item was presented to the left-of-center on the monitor, and each new item appeared to the right of the previous item. The presentation sequence showed a total of seven items. Together, the set of seven items was anchored in the middle of the monitor. Once all seven items were visible, each of the seven items received a text cue to ‘INCLUDE’ or ‘DISCARD’ that appeared above the item. To strengthen the manipulation and the narrative of including/excluding items from a basket, a black rectangle also appeared that outlined the included items, with accompanying black text stating ‘Your basket’. Any discarded items were not included within the boundaries of this outlined basket box (i.e., the discarded item, if any, was instead visible outside the basket’s boundaries; Fig. 3). Once the stimulus set was fully presented, participants could click a 9-point rating scale (1 = not environmentally friendly, 9 = very environmentally friendly) to give a response and end a trial.

Regarding experimental design, Experiment 2 was a factorial design with three within-participants factors: color (green and red), sequence type (primacy and recency), and cue (include and discard). These three factors were also used in Experiment 1, but we omitted the fourth factor ‘sequence length’ since it had no influence on the context effect or the recency effect in Experiment 1. Exactly one item in each sequence had an eco-label (Color: green or red), except for control sequences. Sequence type determined whether the eco-labeled item would appear first (primacy) or last (recency) in the sequence. Cue determined whether all seven items would be included (include) or whether all six non-labeled items would be included and the eco-labeled item would be discarded (discard). We also added two control conditions which had no eco-labeled items; one condition where all items were included; and one condition where the first item was discarded. There were a total of 10 conditions. Each condition was repeated four times, totaling 40 trials. The dependent variable was the mean ratings of the four trials per condition. The order of conditions was randomized, and we also ensured that all ten conditions were completed before any condition would be repeated. At the start of the experiment, participants completed three practice trials. Participants were debriefed upon completion.

## Results and discussion

**Fig. 4:**
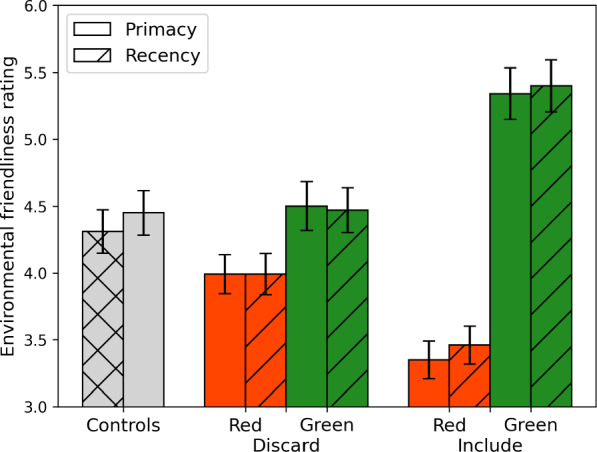
Results of Experiment 2: Data are split across the three experimental factors. Primacy and recency refer to where in the stimulus sequence, the eco-labeled item (red/green) and its cue (discard/include) was placed. In the left-most control condition, all seven items were included in the basket. The right-most control condition had a primacy cue to discard, but the discarded item was not eco-labeled. Error bars represent the standard errors of the means

As can be seen in Fig. 4, the conditions with discarded green and red items did not produce the same contrast effect as previously seen in Experiment 1 (Fig. 2). Instead, discarded green items produced a higher rating of environmental friendliness than discarded red items—an assimilation effect. The assimilation effect shows that, despite the cues to discard the eco-labeled products, the discarded products were considered together with the basket, pulling the environmental valence of the basket toward the environmental valence of the discarded eco-labeled items. This assimilation effect occurred similarly for both primacy,* t*(73) = 3.25, *p* = .002, *d*_z_ = .378, Mean difference = 0.507 (95% CI [0.196–0.817]), BF_10_ = 15.18, and recency sequences,* t*(73) = 3.14, *p* = .002, *d*_z_ = .365, Mean difference = 0.48 (95% CI [0.175 – 0.784]), BF_10_ = 11.13. Bayes factors strongly favored the hypothesis over the null hypothesis. We return to discuss possible explanations as to why the two experiments produced contrast and assimilation effects, respectively, in the general discussion.

As shown in Fig. 4, comparisons with the control conditions show that the effect of assimilation with discarded items was present for red but not green items. Discarded red items (primacy) differed significantly from the matched control condition (primacy), *t*(73) = 3.43, *p* < .001, *d*_z_ = .399, mean difference = 0.456 (95% CI [0.133–0.721]), BF_10_ = 25.01, and the Bayes factor strongly favored the hypothesis over the null hypothesis. But discarded green items (primacy) did not significantly differ from the matched control condition (primacy), *t*(73) = −0.4, *p* = .689, *d*_z_ = −.047, mean difference = -0.051 (95% CI [−0.302–0.201]), BF_10_ = 0.138, and the Bayes factor moderately favored the null hypothesis over the hypothesis. This asymmetry indicates that the assimilation effect was mainly activated by negative environmental valence (red eco-label), broadly consistent with the negativity bias (e.g., Rozin & Royzman, 2001).

Of secondary interest, we also analyzed the full repeated measures design with an omnibus ANOVA, with factors 2 (Color: green and red) × 2 (Sequence type: primacy and recency) × 2 (Cue: include and discard). The results (Fig. 4) revealed the expected main effect of color, *F*(1, 73) = 98.62, *p* < .001, *η*^2^_*p*_ = .575, BF_10_ = 1.462 × 10^14^, and interaction between color × cue, *F*(1, 73) = 42.88, *p* < .001, *η*^2^_*p*_ = .370, BF_10_ = 1.006 × 10^6^, but no other main effects or interactions. There was thus no evidence of any sequence type effects (Fig. 4), suggesting that product sequences that remain visible do not produce primacy or recency effects in judgments. This was expected as primacy and recency effects in judgments likely reflect serial position effects in memory (e.g., Sörqvist et al., 2025), and Experiment 2 did not require memory of the items since they remained visible during judgment.

## General discussion

The results of the present study highlight the temporal dynamics of judgment formation. When each item disappeared after presentation, a contrast effect from item exclusion was revealed: Discarding a red (environmentally harmful) product increased the perceived environmental friendliness of the remaining set in the basket, while discarding a green (environmentally friendly) product decreased it (Experiment 1). In turn, when each item remained visible after presentation, an assimilation effect was revealed: Discarding a red (environmentally harmful) product decreased the perceived environmental friendliness of the included set in the basket, while discarding a green (environmentally friendly) product increased it, even though the content of the to-be-estimated items in the basket was the same (Experiment 2).

Our results appear to follow the inclusion/exclusion model (Bless & Schwarz, 2010) but also extend this model by showing that the effect of an explicit exclusion cue does not necessarily produce a contrast effect. One interpretation of the results of Experiment 1 (showing a contrast effect) is that discarding an item triggers a subtraction process, whereby the valence of the discarded item is mentally removed from the judgment of the remaining set. In Experiment 1, removing a red, environmentally harmful item led to an upward adjustment in perceived basket sustainability, while removing a green item led to a downward adjustment. An alternative account to the contrast effect observed in our study is the comparison-based contrast effect (Bless & Schwarz, 2010). This mechanism suggests that an item’s evaluation is relative to its immediate context rather than stemming from a subtraction process. For example, an eco-friendly item appears even more environmentally friendly if preceded by an environmentally harmful item and less so if preceded by another eco-friendly item (Tanner, 2008). Such effects reflect broader temporal dynamics in judgment formation, where contrast effects push judgments away from preceding stimuli, while assimilation effects pull judgments toward concurrent stimuli (Tanner, 2008; Wedell et al., 1987).

A central design feature of the present study was the explicit discard cue. A theoretical implication of the contrast effect from explicit exclusion in Experiment 1 and the assimilation effect from explicit exclusion in Experiment 2 is that exclusion from a to-be-estimated set is not enough to produce a contrast effect. It appears as if the excluded item must also be out of sight. Even with an explicit categorization of items into targets and distracters, incidental comparisons might still produce assimilation if the excluded items are (still) present at the time of judgment (see also Skog et al., 2026a). If subtraction-by-exclusion were responsible for the contrast effect, then contrast effects would be expected from explicit categorization regardless of the to-be-ignored item’s visibility. The fact that an assimilation rather than contrast effect was found even with explicit exclusion in Experiment 2 seems to be more in line with the incidental comparison-based mechanism. Taken together, the results are more consistent with a comparison-based mechanism than with a simple subtraction process.

Another possible explanation for the contrast effect is that exclusion alters how participants use the response scale (Eiser, 1990; Frederick & Mochon, 2012; Wedell, 1996). According to this scale-adjustment hypothesis, discarding a red item raises the response anchor, making subsequent ratings more environmentally friendly, whereas discarding a green item lowers the anchor, leading to less favorable ratings. If this response-anchoring mechanism was the sole explanation, one would expect stronger contrast effects (in Experiment 1) when the first item in the sequence is excluded. This is because an early exclusion would establish an initial judgmental frame, which subsequent items would be assessed against. Similarly, we would expect stronger assimilation effects of green/red items when this to-be-excluded item is presented first in the unfolding sequence (Experiment 2). This is because participants would have more time to prepare a response that is consistent with the green/red item when it is presented first, and less time when the green/red item is presented last. However, our findings do not support this prediction. An important feature of our design was the temporal position of the explicitly excluded item. The excluded item was always presented either before or after the included (to-be-estimated) items. This temporal manipulation neither modulated the contrast effect (Experiment 1) or the assimilation effect (Experiment 2). Contrast effects were just as strong when exclusion occurred at the beginning or at the end of the sequence (Experiment 1, Fig. 2) and assimilation effects were just as strong when the first and the last item was excluded (Experiment 2, Fig. 4). This suggests that assimilation and contrast effects are not simply a function of response scale adjustments but instead depend on whether excluded items are integrated into or segregated from the target basket during judgment.

Finally, our results indicate that the recency effect found in several previous studies (e.g., Aldrovandi et al., 2015; Sörqvist et al., 2024) and the contrast effect found here operate through distinct cognitive mechanisms. In Experiment 1, in which participants had to remember which items to consider for the upcoming judgment after sequence presentation, there was a recency effect of included items. That is, shopping sequences where to-be-included green items appeared at the end were rated as more environmentally friendly, compared to sequences where the same item was positioned at the beginning. And correspondingly, shopping sequences where to-be-included red items appeared at the end were rated as less environmentally friendly, compared to sequences where the same item was positioned at the beginning. The contrast effect of Experiment 1 was comparable in magnitude regardless of whether it occurred from discarding items at the beginning or at the end of the sequence. On the other hand, included items consistently showed a recency effect, exerting the strongest influence when they appeared last, reinforcing previous findings on memory-driven valuation biases (Aldrovandi et al., 2015; Kahneman et al., 1993; Montgomery & Unnava, 2009; Sörqvist et al., 2025). The recency effect likely emerges because the final item is more temporally distinct, enhancing its salience in memory (Sörqvist et al., 2024, 2025). Conversely, contrast effects appear not to depend on such saliency mechanisms, as excluded items produced contrast effects when occluded from sight regardless of temporal position (Experiment 1) and they produced assimilation effects when still visible (and thus salient) at the time of judgment (Experiment 2).

Taken together, the present findings suggest that environmental judgments are influenced not only by the objective composition of a choice set, but also by how excluded information is represented. When discarded items are no longer perceptually available, they appear to function as external comparison standards, producing contrast. When they remain visible, they are more likely to be integrated into the representation of the set, producing assimilation. This distinction highlights the importance of representational format in influencing environmental judgment.

## Limitations

The present study provides evidence that previously seen discarded items can produce contrast and co-present discarded items can produce assimilation. While this finding is supported by theory (e.g., Bless & Schwarz, 2010) and past findings (Tanner, 2008; Wedell et al., 1987), the present study involves methodological differences between Experiments 1 and 2 that may complicate inference. As demonstrated in our two trial illustrations (Fig. 1 and 3), there were differences in the stimulus set-up. Some differences in stimulus procedure were necessary to introduce simultaneous presentation of all products in the basket, but future studies can consider other ways to introduce this change. The replacement of the sequence length factor (Experiment 1) with sequences that were always seven in length (Experiment 2) introduces a difference in the stimulus procedure which we cannot rule out as a confound. Finally, control conditions were used in the pilot experiment (which used a sequential presentation format), but were then omitted in Experiment 1, then reintroduced in Experiment 2. This changes the eco-label predictability between Experiment 1 and Experiment 2, where participants in Experiment 1 might incidentally learn that an eco-label will always be present in each trial, whereas 20% of trials had no eco-label in Experiment 2. The absence of a control condition also prevents an analysis of a baseline for Experiment 1, which limits the ability to infer whether the contrast effect was driven by the green, red, or both eco-labels.

## Concluding remarks

This study provides evidence that discarded products can systematically shift how consumers perceive the environmental impact of their purchases. Our findings suggest that discarding an item with a high carbon footprint can enhance perceived environmental friendliness, while discarding an eco-friendly item diminishes it, but only when the discarded item is out of sight. Conversely, when the discarded item remains in sight, discarding an item with a high carbon footprint decreases the perceived environmental friendliness of the remaining items, while discarding an eco-friendly item can enhance it.

These cognitive biases have real-world implications: Environmentally conscious consumers tend to actively avoid purchasing high carbon footprint products (Gao & Souza, 2022), and our results suggest that this exclusion process may inflate their perception of their own eco-consciousness. A critical next step for future research is determining whether these perceptual shifts translate into behavioral consequences. If discarding high carbon products enhances perceived environmental responsibility, it may reduce motivation to make further sustainable choices—an effect consistent with rebound effects (e.g., Seebauer, 2018) or moral licensing (Mazar & Zhong, 2010). Conversely, consumers who discard eco-friendly products may downplay their environmental impact, reinforcing biases against sustainable consumption. This suggests that consumer decision making is not only influenced by purchased products but also by rejected products. Future studies should examine whether contrast-driven shifts in perception influence real-world purchasing decisions, particularly in contexts where consumers make ethical trade-offs (e.g., organic vs. conventional goods). If contrast effects systematically distort self-perceptions of sustainability, they could have important implications for interventions aimed at encouraging pro-environmental behavior.

## Supplementary Information

Additional file1 (DOCX 459 KB)

## Data Availability

All data and materials are available on the Open Science Framework (OSF; 10.17605/OSF.IO/XH5EA).
