# Supplementary material for: Out of sight, out of mind? How discarded items shape environmental judgments
Source: Cogn Res Princ Implic. 2026 Jun 28;11:37. doi: 10.1186/s41235-026-00743-9 (PMC13315070; doi:10.1186/s41235-026-00743-9)
Supplement: Supplementary file 1 — Additional file1 (DOCX 459 KB) [file 41235_2026_743_MOESM1_ESM.docx]

**Pilot experiment**

Discarding an item may alter the perceived environmental friendliness of the basket, even though its contents remain unchanged. If a ‘high carbon footprint’ label is *included* in the basket, we expected a less favorable environmental evaluation, especially when this item appears at the end of the shopping sequence (i.e., a recency effect). However, based on the inclusion/exclusion model of assimilation and contrast (Bless & Schwarz, 2010), we predict a reversed effect when such an item is *excluded*: Discarding a high carbon footprint product should make the remaining selection appear more environmentally friendly, whereas discarding an eco-friendly item should have the opposite effect, making the basket seem less environmentally friendly. In the pilot experiment, we tested these hypotheses by examining whether judgements of environmental impact were modulated by 1) whether an item was included or discarded, and 2) the item’s sequential position in the shopping sequence (e.g., beginning, middle, or end). The trial structure in the pilot experiment took inspiration from the item-wise procedure of the directed forgetting literature (Basden, 2010; MacLeod, 1999), where sequential items are presented with cues to include or discard.

**Method**

***Participants***

Sixty participants (31 female, 29 male, Mean age = 45.9 years, *SD* = 14.8) took part in this pilot experiment. Participants were recruited via Prolific ([www.prolific.com](http://www.prolific.com)), with the exception of one participant who was a student at the University of Central Lancashire. All participants were UK-based, native English speakers with normal or corrected-to-normal vision and no reported color-blindness. The sample size was determined through a power analysis using G*Power (Faul et al., 2007). We assumed that the expected contrast effect for this pilot experiment (with the purpose of preparing and planning for Experiment 1) would be of medium size (Cohen’s *d*_z_ = 0.35) and set power (1 – β error probability) at .80, resulting in a required sample size of *N* = 59. This estimate was informed by previous studies demonstrating robust recency effect in eco-labeling with similar sample sizes (Sörqvist et al., 2024a). To ensure sufficient statistical power, we collected data from 60 participants. Ethical approval was granted by the Psychology and Social Work Ethics Committee at the University of Central Lancashire (Approval code: 507), and the work was conducted in accordance with the Declaration of Helsinki and Swedish Research Ethics Authority guidelines (Dnr 2024-05795-01). Participants were compensated at a rate of £9 per hour for a total study time of approximately 23 minutes.

***Materials***

The experiment was programmed in PsychoPy (version 2024.1.0, Peirce et al., 2019) and deployed online using Pavlovia. Participants accessed the experiment via a Prolific URL and were required to use a desktop computer.

Stimuli consisted of 81 images of grocery store products (e.g., packages of rice, meat, fruit). Each image was displayed above a text label describing the item (e.g., ‘1 bunch of bananas’). The items were used to construct seven sequence types: Control sequences (items without eco-labels), green eco-label sequences (where a green-labelled item appeared in the first, middle, or last position), and red eco-label sequences (where a red-labelled item appeared in the first, middle or last position). Each sequence contained five items, and item selection was randomized on each trial to prevent systematic pairing biases between specific products and eco-labels.

***Design and procedure***

A within-participants design was used. The study was advertised on Prolific as an investigation of ‘Environmental impact and grocery shopping’. Before starting the experiment, participants read a participant information sheet, provided informed consent, and received detailed task instructions. They were informed that they would view sequences of grocery store products, with some produce labelled green (eco-friendly) or red (high carbon footprint). Participants were instructed that each product would be followed by a cue indicating whether it should be included or discarded from their shopping basket. They were told only to consider the included items when making environmental impact judgements. Participants were explicitly instructed to evaluate each shopping basket independently, without considering the items shown during previous trials.

Each trial began when participants pressed the spacebar. Thereafter, a sequence of five items was presented one at a time in the center of the screen. Each item was displayed for 1000 ms, followed by a 200 ms blank interval. A cue ‘INCLUDE’ or ‘DISCARD’ then appeared for 1000 ms, followed by another 200 ms blank interval before the next item. The total sequence duration was 12 seconds before the judgment phase.

After viewing the sequence, participants rated the environmental friendliness of their shopping basket using a 9-point scale (1 = not environmentally friendly, 9 = very environmentally friendly). Participants then pressed the spacebar to initiate the next trial. Upon completion, they were presented with a debrief form explaining the study’s objectives.

Each sequence contained either one discard cue or none, with the remaining items always marked for inclusion. Discard cues were positioned at either the first, middle, or last item in the sequence. If a sequence included a red or green eco-labelled item, the discard cue was always paired with this item. Control sequences (without eco-labels) followed four patterns: all items included, or a discard cue after the first, third, or fifth item. The experiment employed a 2 (Color: green, red) × 2 (Memory cue: include, discard) × 3 (Sequence type: primacy, middle, recency) factorial within-participants design, comprising 16 conditions (including controls). Each condition was presented four times, totaling 64 trials per participant. Presentation order was randomized, ensuring all conditions were seen before any repetition occurred. Before the main part of this pilot experiment, participants completed three practice trials to familiarize themselves with the task. These trials were not analyzed.

**Results and discussion**

Our analyses examined the effects of eco-label color (green, red), memory cue (include, discard) and sequence position (primacy, middle, recency) on participants’ environmental friendliness ratings. Results are summarized in Figure S1. Data from the pilot experiment are publicly available online at the Open Science Framework (OSF; <https://doi.org/10.17605/OSF.IO/XH5EA>). The results of the pilot experiment provided an initial estimate of the hypothesised contrast effect, in which baskets (i.e., stimulus sequences) were judged as more environmentally friendly when a red item was discarded during sequence presentation, compared to when a green item was discarded. A repeated measures 2 (Color: green vs red) × 2 (Sequence type: primacy vs recency) × 2 (Cue: include vs discard) analysis of variance found a significant three-way interaction, *F*(1, 59) = 5.25, *p* = .026, η^2^_p_ = .082, BF_10_ = .506, and a paired samples *t*-test on the corresponding difference scores yielded an effect size of *d*_z_ = .296, classified as small-medium. However, the Bayes factor anecdotally favored the null hypothesis over the hypothesis, indicating that the result was statistically unreliable. We note further that this three-way interaction was not significant when it considered the full design, including ‘middle’ sequence types. The pilot study did not provide clear evidence for a contrast effect, yet it was sufficient to gain an estimate of the hypothesized contrast effect (under conditions where the ‘middle’ sequence type was omitted), and it established the general experimental procedure that Experiment 1 was based on.


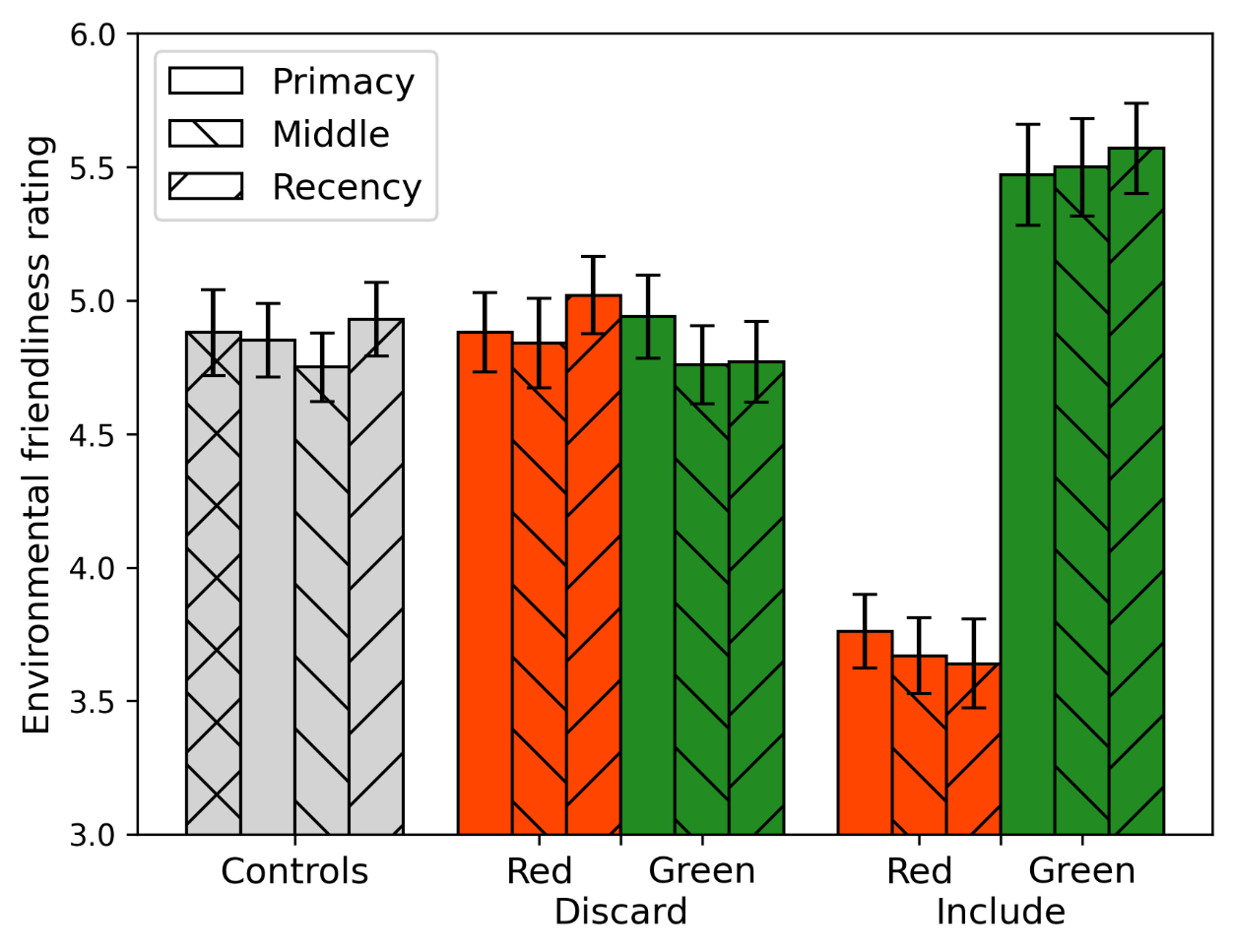


*Figure S1.* Results of pilot experiment. Data are split across the three experimental factors. Primacy, middle and recency refer to where in the stimulus sequence the eco-labelled item (red/green) and its cue (discard/include) was placed. In the left-most control condition, all five items were included in the basket. Error bars represent the standard errors of the means.
